# Clinically Relevant Dimer Interface Mutants of STAT1 Transcription Factor Exhibit Differential Gene Expression

**DOI:** 10.1371/journal.pone.0069903

**Published:** 2013-07-26

**Authors:** Julia Staab, Christoph Herrmann-Lingen, Thomas Meyer

**Affiliations:** Department of Psychosomatic Medicine and Psychotherapy, University of Göttingen, Göttingen, Germany; University of Regensburg, Germany

## Abstract

A transition from a parallel to an antiparallel dimer configuration of the transcription factor *s*ignal *t*ransducer and *a*ctivator of *t*ranscription 1 (STAT1) is required for interferon (IFN)-mediated signal transduction. However, the precise molecular mechanisms linking conformational changes to target gene activation by STAT1 are still largely unknown. In the present study, we have characterized, in more detail than before, two disease-associated point mutants with amino acid substitutions at both sites of the dimer interface (F172W and T385A). First, we confirmed that IFNγ-stimulation of transfected cells led to enhanced tyrosine phosphorylation of mutant STAT1 as compared to the wild-type protein, which consequently resulted in its prolonged nuclear accumulation. Using an *in vitro* dephosphorylation assay, we demonstrated that, in contrast to wild-type STAT1 and similar to the F172W mutant, also T385A resisted enzymatic inactivation by the nuclear phosphatase Tc45. Transcriptional activation of IFNγ-driven endogenous target genes differed between wild-type and mutant STAT1. While expression of genes containing a single classical gamma-activated site (GAS), such as *irf1*, *gpb1*, and *mig1*, was virtually unaffected by the presence of either of two amino acid exchanges, induction of the c*xcl10* and *mcp1* gene was significantly enhanced. The latter two genes both contain an additional TTC/GAA binding motif separated by 10 bp from the palindromic GAS sequence. The transcriptional superiority of the mutants on these genes was reflected by their increased binding affinity to DNA fragments containing the identified “one-and-a-half-GAS” motif. In summary, our data demonstrate that two clinically relevant interface mutants of STAT1 exhibit gene-specific effects and point to the rather complex role of the assumed conformational shift between two different dimer configurations for efficient transcriptional regulation.

## Introduction

Transcriptional regulation by *s*ignal *t*ransducer and *a*ctivator of *t*ranscription 1 (STAT1) plays a crucial role in the control of immune reactions and represents a major line of defense against invading microorganisms. The importance of STAT1 was clearly established by the generation of knockout and knockin mouse lines, the latter carrying a substitution of serine 727, which are highly susceptible to viral and bacterial infections [1]–[3]. In patients, several germline mutations in the human STAT1-encoding gene have been identified as the genetic causes of autosomal dominant chronic mucocutaneous candidiasis (CMC) and Mendelian susceptibility to mycobacterial disease (MSMD) [4]–[14]. The majority of mutations in CMC patients were found in the STAT1 coiled-coil domain [9], [10], whereas recently published papers reported on a substitution of methionine for threonine in the DNA-binding domain (T385M), which was detected in two unrelated Japanese CMC patients [11] and a 21-year-old white male with severe disseminated histoplasmosis [14]. All CMC patients with disease-associated mutations in the *stat1* gene suffered from symptoms of immunodeficiency, including chronic and recurrent *Candida* infections of the skin, nails, and oropharynx, while the two T385M carriers additionally developed bronchiectasis in their early childhood [11]. Sampaio et al. recently detected the STAT1-F172L mutation in a 25-year-old woman with recurrent oral, cutaneous, and vaginal candidiasis and a history of disseminated histoplasmosis [14]. These authors also reported on a T385M substitution in two patients with FOXP3 wild-type immune dysregulation-polyendocrinopathy-enteropathy-X-linked-like syndrome [15].

From these novel clinical observations, and a variety of previous studies, it has been well established that STAT1 functions as a transcription factor in regulating interferon (IFN)-mediated immune responses [16]–[20]. The intracellular processing of IFN signals entails the cytoplasmic activation and nuclear accumulation of STAT1 homo- or heterodimers. Engagement of IFNs with their cognate receptors at the cell membrane triggers autophosphorylation on tyrosines of non-covalently attached JAK kinases, which also phosphorylate signature tyrosine residues in the intracellular receptor tails [21]. Cytoplasmic STAT1 molecules are then recruited to the ligand-bound IFN receptors via their Src homology 2 (SH2) domains, where they are phosphorylated by JAK kinases on a single tyrosine residue at their carboxy-terminus [22]–[25]. Phospho-STAT1 dimers are translocated into the nucleus via importin-α/β-mediated transport, where they recognize the palindromic consensus sequence 5′-TTCN_2–4_GAA-′3 that defines an IFNγ-activated site (GAS) [26]–[28]. In addition, unphosphorylated STAT1 constitutively shuttles between the cytoplasm and the nucleus through direct contacts with nucleoporins located in the nuclear pore complex [29], [30].

As opposed to the antiparallel dimeric structure of non-phosphorylated STAT1, activation of STAT1 is associated with the emergence of a parallel dimer conformation formed by reciprocal interactions between the phospho-tyrosine residue 701 on one monomer and the SH2 domain on the partner monomer [31]–[33]. For GAS recognition, the SH2 domains of two monomers have to be oriented at one end of the dimer, with the DNA separating the monomers along the long axis of the STAT1 core molecule [34]. In contrast, in the crystal structure of the antiparallel conformation, the two SH2 domains are located at opposite ends of the dimer, with the phenylalanine residue 172 in the coiled-coil domain of one monomer interacting reciprocally with a pocket structure in the DNA-binding domain of its partner monomer [35].

While there is growing evidence in support of a conformational shift between the two dimer configurations, much less is known on how this transition might affect cytokine-driven gene expression. In the present paper, we have characterized two of the recently identified disease-causing STAT1 mutations in more detail with regard to gene activation. The differential gene expression profile associated with these mutations allowed us to draw conclusions about the impact of the supposed dimer transition on transcriptional activity.

## Materials and Methods

### Cell Culture

All cell lines used in this study were cultured in a humidified 5% CO_2_ atmosphere. HeLa cells were grown in Quantum 101 media (PAA Laboratories) supplemented with 1% penicillin/streptomycin (PAA Laboratories). STAT1-negative U3A cells were cultured in Dulbecco’s modified Eagle’s medium (DMEM, Biochrom) containing 10% FCS (Biochrom) supplemented with antibiotics [36]. Transfection was achieved with MegaTran1.0 (Origene) according to the manufacturer’s recommendation. Twenty-four hours after transfection, cells were either left unstimulated or stimulated with 5 ng/ml IFNγ (Biomol) for 45 min. Subsequently, cells were treated with 1 µM staurosporine (Sigma) for the indicated times. In some experiments, IFNγ-stimulated cells were additionally treated for 30 min with 0.8 mM sodium vanadate and 0.2 mM H_2_O_2_.

### Plasmids

The plasmid pSTAT1-GFP coding for full-length human STAT1 (amino acids 1–746) fused carboxyterminally to green fluorescent protein (GFP) has been described [37]. For immunocytochemistry, gelshifts and real-time PCR, U3A cells were transfected with a pcDNA3.1 expression vector (Invitrogen) coding for untagged human STAT1. The substitution of alanine for threonine in position 385 (T385A) and tryptophan for phenylalanine 172 (F172W) was generated in the two STAT1-coding plasmids pEGFP-N1 and pcDNA by site-directed point mutagenesis using the QuikChange® II kit from Stratagene according to the manufacturer’s recommendation. All mutations were confirmed by DNA sequencing (SeqLab).

### Fractionated Cell Extraction and Western Blotting

STAT1-expressing cells grown on 6-well dishes were lysed in 50 µl cytoplasmic extraction buffer (20 mM Hepes, pH 7.4, 10 mM KCl, 10% (v/v) glycerol, 1 mM EDTA, 0.1 mM Na_3_VO_4_, 0.1% IGEPAL-CA-360, 3 mM DTT, 0.4 mM Pefabloc, Complete Mini protease inhibitors (Roche)) for 5 min on ice. After centrifugation at 13.000 rpm for 15 sec at 4°C, the supernatants were spun again for 5 min at 13.000 rpm and 4°C and the supernatants resulting from this step were collected as cytoplasmic extracts. The pellets from the first centrifugation were lysed in 50 µl nuclear extraction buffer (20 mM Hepes, pH 7.4, 420 mM KCl, 20% (v/v) glycerol, 1 mM EDTA, 0.1 mM Na_3_VO_4_, 3 mM DTT, 0.4 mM Pefabloc, and Complete Mini protease inhibitors) for 30 min on ice. After centrifugation at 13.000 rpm for 15 min and 4°C, supernatants were collected as nuclear extracts. For whole cell extracts, the same amount of cytoplasmic and nuclear extracts were mixed and boiled in SDS sample buffer. Proteins were then resolved by 10% SDS-PAGE and transferred to PVDF membranes. The membranes were incubated first with a polyclonal phospho-STAT1-Tyr701-specific antibody (Cell Signaling) followed by incubation with a conjugated secondary antibody (LI-COR). To determine the amount of total STAT1, blots were stripped at 60°C for 60 min in a buffer containing 2% SDS, 0.7% β-mercaptoethanol, and 62.5 mM Tris-HCl, pH 6.8 and subsequently reprobed with the STAT1-specific polyclonal antibody C-24 (Santa Cruz Biotechnology). Bound immunoreactivity was detected with anti-rabbit IRDye 800CW antibodies visualized on a LI-COR Odyssey imaging machine.

### *In vitro* Dephosphorylation

For *in vitro* dephosphorylation, STAT1-reconstituted U3A cells were treated for 45 min with IFNγ (5 ng/ml) before whole cell extracts were prepared. Ten µl of each extract were mixed with the same volume of dephosphorylation buffer (25 mM Tris-HCl, pH 7.5, 0.5 mg/ml BSA, 50 mM KCl, 5 mM EDTA, 20 mM DTT, Complete Mini protease inhibitors) containing 2 units of T-cell protein tyrosine phosphatase (Tc45-PTP, Enzo Life Sciences). Samples were incubated at 30°C for the indicated times and the dephosphorylation reactions were stopped by adding 6x SDS sample buffer. The ratio of tyrosine-phosphorylated to total STAT1 was tested by means of Western blotting, as described above.

### Fluorescence Mircroscopy

For direct microscopical detection of GFP-tagged STAT1, transiently transfected HeLa cells cultured on cover slips were treated subsequently with IFNγ and staurosporine as indicated. Cells were fixed with 4% paraformaldehyde in phosphate-buffered saline (PBS) for 15 min at room temperature (RT). Nuclei were stained with 5 µg/ml Hoechst 33258 (Sigma) for 10 min at RT and the samples were mounted in fluorescence mounting medium (Southern Biotech). Fluorescence microscopy was performed using a Leica DM5000B microscope equipped with appropriate fluorescence filters. Images were obtained with a CCD camera and further processed with the Leica QWin software.

### Immunocytochemistry

U3A cells were seeded on 8-well chamber slides for 24 h before being transfected with a pcDNA expression vector coding for either wild-type or mutant STAT1. After IFNγ stimulation and treatment with staurosporine for the indicated times, cells were fixed with methanol for 15 min at −20°C. After having been permeabilized for 20 min in 1% Triton X-100/PBS and the blocking of non-specific binding with 25% FCS/PBS for 45 min at RT, samples were incubated with the anti-STAT1 antibody C-24 (Santa Cruz) diluted 1∶1000 in 25% FCS/PBS for 45 min at RT. Following repeated washing in PBS, cells were incubated for 45 min at RT with Cy3-conjugated secondary antibody (Dianova) diluted 1∶500 in 25% FCS/PBS followed by nuclear staining with Hoechst dye. Finally, the samples were mounted and images were captured by fluorescence microscopy as described above.

### Electrophoretic Mobility Shift Assay (EMSA)

The cells were stimulated for 45 min with IFNγ, then treated with staurosporine, and whole cell extracts were prepared as described above. Cellular extracts (4.5 µl) were equilibrated for 15 min at RT with 1 ng [^33^P]-labeled duplex oligonucleotide probe, which was generated by an end-filling reaction using Klenow fragment (New England Biolabs). The following duplex oligonucleotides were used (GAS sites and TTC/GAA motifs are underlined; the respective antisense oligos and 5′ poly-(T)_6_ overhangs used for labeling are not listed):

M67 5′-CGACATTTCCCGTAAATCTG-3′, 2xGAS 5′-CGTTTCCCCGAAATTGACGGATTTCCCCGAAAC-3′, GAS-nonGAS 5′-CGTTTCCCCGAAATTGACGGATTTACCCCAAC-3′, 2xnonGAS 5′-CGTTTACCCCAAATTGACGGATTTACCCCAAC-3′, MCP1-A: 5′-CTGCTAGCCTTTCCTACTTCCTGGAAATCCA-3′, MCP1-B: 5′-CTGCTTCCCTTTCCTACTTCCTGGAAATCCA-3′, MCP1-C: 5′-CTGCTTCCCTTTCCTACTAGCTGGAAATCCA-3′,

To assess dissociation rates of the STAT1 variants from DNA, a 750-fold molar excess of unlabeled M67 DNA was incubated with the shift reactions for the indicated times. Subsequently, the reactions were separated on a native 4.8% acrylamide:bisacrylamide gel (29∶1) at 4°C. Binding activity was visualized with a phosphoimaging system (Fujifilm FLA-5100, Fuji) using the computer programs Aida Image Analyzer v. 4.06 and TINA version 2.0 (Raytest). In supershift reactions, 20 ng of the STAT1-specific antibody C-24 or the STAT3-specific antibody H-190 (both from Santa Cruz Biotechnology) were preincubated with the shift reactions for 20 min at RT.

### Reporter Gene Assay

For reporter gene assays, U3A cells grown on 48-well plates were co-transfected in each well with the following three plasmids: 70 ng of a luciferase reporter, 200 ng of a constitutively expressed β-galactosidase plasmid, and 250 ng of a pSTAT1-GFP plasmid expressing either wild-type or mutant STAT1. The reporter construct contained a triple Ly6E STAT-binding site (termed 3xLy6E) upstream of the transcription start site of the luciferase gene [38]. Before whole cell extracts were prepared, cells were either left unstimulated or stimulated for 6 h with IFNγ followed by staurosporine treatment for the indicated times. In each sample, luciferase expression was assessed (Promega) and normalized to the corresponding β-galactosidase activity, which was measured spectroscopically at 420 nm. For each STAT1 variant and stimulation mode, six independent transfections were tested and the experiment was repeated at least in triplicate.

### Real-time PCR

Transfected U3A cells expressing either wild-type or mutant STAT1 were cultured for 15 h with DMEM supplemented with 1% FCS. Cells were then either left untreated or stimulated with 5 ng/ml IFNγ for 6 h. RNA was isolated using the peqGold Total RNA kit (peqlab) and cDNA was synthesized using the Verso cDNA kit (Thermo Scientific) according to the manufacturers’ recommendations. Each real-time PCR reaction was measured in duplicate in a total volume of 20 µl, containing 1 µl of cDNA, 70 nmol/l of each primer, and 10 µl of Absolute Blue QPCR SYBR Green mix (Thermo Scientific). Gene-specific primers for five endogenous IFNγ-inducible cDNAs as well as for *stat1* and *gapdh* were obtained from Sigma-Aldrich. The following primer pairs were used in this study: CXCL10F 5′-ATTCTGAGCCTACAGCAGAG-′3, CXCL10R 5′-GCTTGCAGGAATAATTTCAA-′3, GAPDHF 5′-GAAGGTGAAGGTCGGAGTC-′3, GAPDHR 5′-GAAGATGGTGATGGGATTTC-′3, GBP1F 5′-GGTCCAGTTGCTGAAAGAGC-′3, GBP1R 5′-TGACAGGAAGGCTCTGGTCT-′3, IRF1F 5′-AGCTCAGCTGTGCGAGTGTA-′3, IRF1R 5′-TAGCTGCTGTGGTCATCAGG-′3, MIG1F 5′-CCACCGAGATCCTTATCGAA-′3, MIG1R 5′-CTAACCGACTTGGCTGCTTC-′3, MCP1F 5′-CCAGTCACCTGCTGTTATAAC-′3, MCP1R 5′-TGGAATCCTGAACCCACTTCT-′3, STAT1F 5′-CCGTTTTCATGACCTCCTGT-′3, and STAT1R 5′-TGAATATTCCCCGACTGAGC-′3.

Samples were amplified using the following PCR protocol: a denaturation step at 95°C for 15 min followed by 40 cycles of denaturation at 95°C for 15 sec, annealing at 55°C for 30 sec, and extension at 72°C for 30 sec. Following the final amplification step, a melting curve analysis was run via a temperature gradient from 60°C to 95°C in 0.5°C increment steps, with fluorescence being measured at each temperature for a period of 10 s. Each mRNA expression was normalized to the expression of *gapdh*. The ΔΔCt method was used to determine comparative relative expression levels, based on the formula 2^–(ΔCt target –ΔCt reference sample)^.

### Statistical Analyses

Means and standard deviations were calculated for each variant and stimulation mode. Differences in DNA binding activity and gene expression between the STAT1 variants were assessed using Student’s *t* tests and Mann-Whitney-Wilcoxon tests, where appropriate. For all analyses, p<0.05 was considered statistically significant.

## Results

### STAT1-T385A is Associated with Elevated Tyrosine Phosphorylation

**Figure 1:**
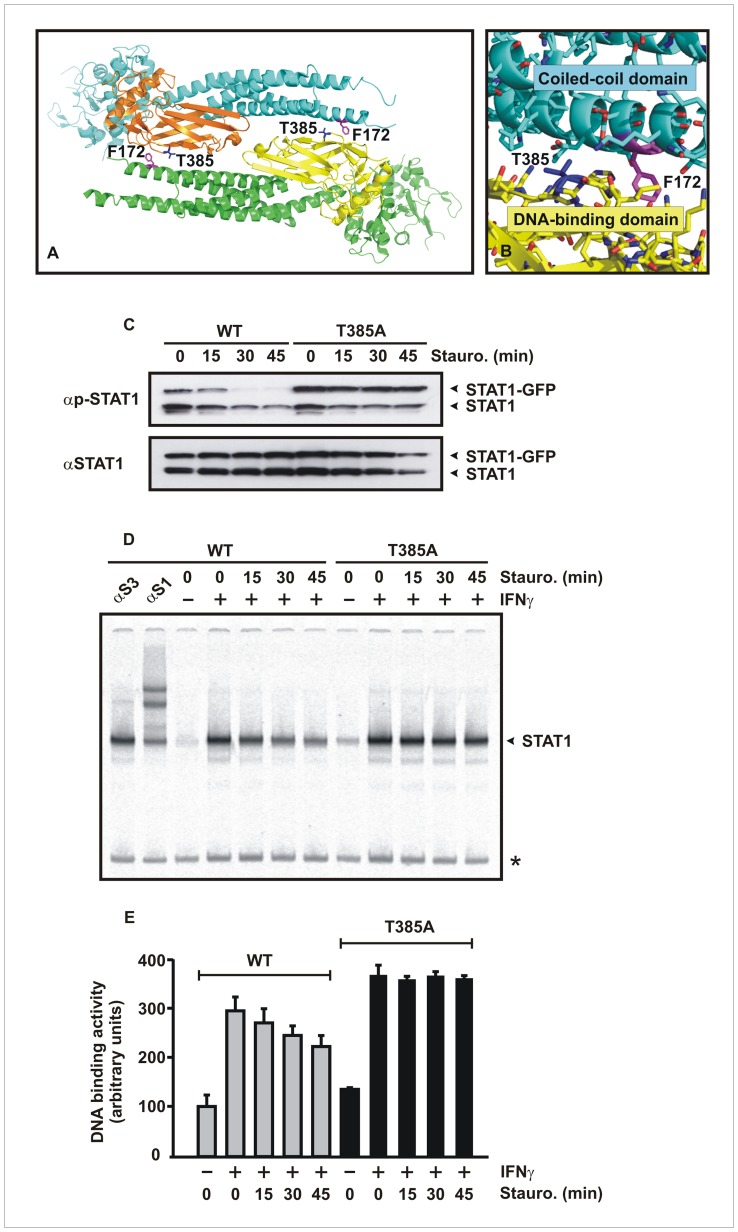
Phenylalanine 172 in the coiled-coil domain and threonine 385 in the DNA-binding domain (the latter colored in orange and yellow) are part of the interface surface formed by two STAT1 monomers oriented in antiparallel alignment. (A) Crystal structure of a truncated STAT1 dimer showing the localization of F172 (in magenta) and T385 (in dark blue), including a closer view of the ribbon representation (B), demonstrates the spatial orientation of the functional groups of F172 and T385, which are required for interaction with the partner monomer in the antiparallel dimer conformation. (C) Substitution of alanine for threonine 385 resulted in prolonged and elevated tyrosine phosphorylation of mutant STAT1 in cells stimulated with interferon-γ. Equal numbers of HeLa cells expressing either wild-type (WT) or mutant STAT1, both tagged with green fluorescent protein (GFP), were stimulated with 5 ng/ml of IFNγ for 45 min, before the kinase inhibitor staurosporine (1 µM) was added for the indicated times. Whole cell extracts were assessed for the time course of tyrosine phosphorylation by means of Western blotting using a STAT1-specific phospho-tyrosine antibody followed by a secondary antibody (αp-STAT1, top panel). The same membrane was stripped off bound immunoreactivity and re-probed with the pan-STAT1 antibody C-24 (αSTAT1, bottom panel). The upper arrowhead marks GFP-tagged STAT1 and the lower one indicates endogenous STAT1. (D, E) Gelshift assays confirm the increased DNA-binding activity of the T385A mutant which resulted from enhanced tyrosine phosphorylation. STAT1-negative U3A cells reconstituted with either wild-type or mutant STAT1 were stimulated with IFNγ (5 ng/ml) and subsequently treated with staurosporine for the indicated times, before whole cell lysates were incubated with [^33^P]-labeled duplex oligonucleotides containing a GAS site (M67) and loaded onto a native polyacrylamide gel. The asterisk at the right-hand margin of the gel marks a non-specific band and the arrowhead corresponds to recombinant STAT1 bound to M67. (E) Time of DNA binding activity of STAT1-WT and T385A to M67 as quantified from EMSA experiments such as in (D).

To functionally characterize the disease-causing threonine 385 mutation in the STAT1 DNA-binding domain, we exchanged this residue for alanine and, in a first set of experiments, compared this mutant to the methionine substitution found in patients with aberrant regulation of IFNγ-mediated inflammation [11], [14], [15]. Threonine 385 is localized at the surface of the DNA-binding domain, where it directly faces the coiled-coil domain of the antiparallel partner monomer, as revealed from the crystal structure of the non-phosphorylated human STAT1 dimer (Fig. 1A). The aromatic side chain of phenylalanine 172, which is required for the stability of the antiparallel dimer configuration [31], [32], [35], is only 5.4 Å away from the threonine residue 385 in the partner monomer (Fig. 1B). Sequence alignment showed that the STAT1 threonine residue 385 is conserved in human STAT3 and STAT4, whereas in STAT2 the homologous position is occupied by a serine.

Immunoblotting experiments confirmed that the T385A mutant showed similarly elevated tyrosine phosphorylation levels as those reported for the methionine substitution [11], [13]. Upon stimulation of cells with IFNγ, the T385A mutant displayed a significantly prolonged time course of tyrosine-phosphorylation as compared to the wild-type molecule (Fig. 1C). When HeLa cells expressing green fluorescent protein (GFP)-tagged STAT1 were pretreated with 5 ng/ml IFNγ for 45 min and subsequently incubated with 1 µM of staurosporine, the GFP-tagged wild-type (WT) STAT1 molecule nearly completely lost its tyrosine phosphorylation signal within 30 min after the addition of the kinase blocker. In contrast, STAT1-T385A-GFP was partially resistant towards the inhibitory effect of staurosporine, as the mutant remained tyrosine-phosphorylated for at least 45 min. Similar results were obtained, when whole cell extracts from IFNγ-pretreated, STAT1-reconstituted U3A cells lacking expression of endogenous STAT1 were tested for their binding activity to a radioactively labeled GAS site by means of electrophoretic mobility shift assays (EMSA, Fig. 1D,E). Again, exposure of the cells to staurosporine resulted in a time-dependent decrease in the DNA-binding activity of wild-type STAT1 after 15 min, whereas no such reduction was observed for the T385A mutant.

### The T385A Alteration Leads to Prolonged Interferon-induced STAT1 Nuclear Accumulation

**Figure 2:**
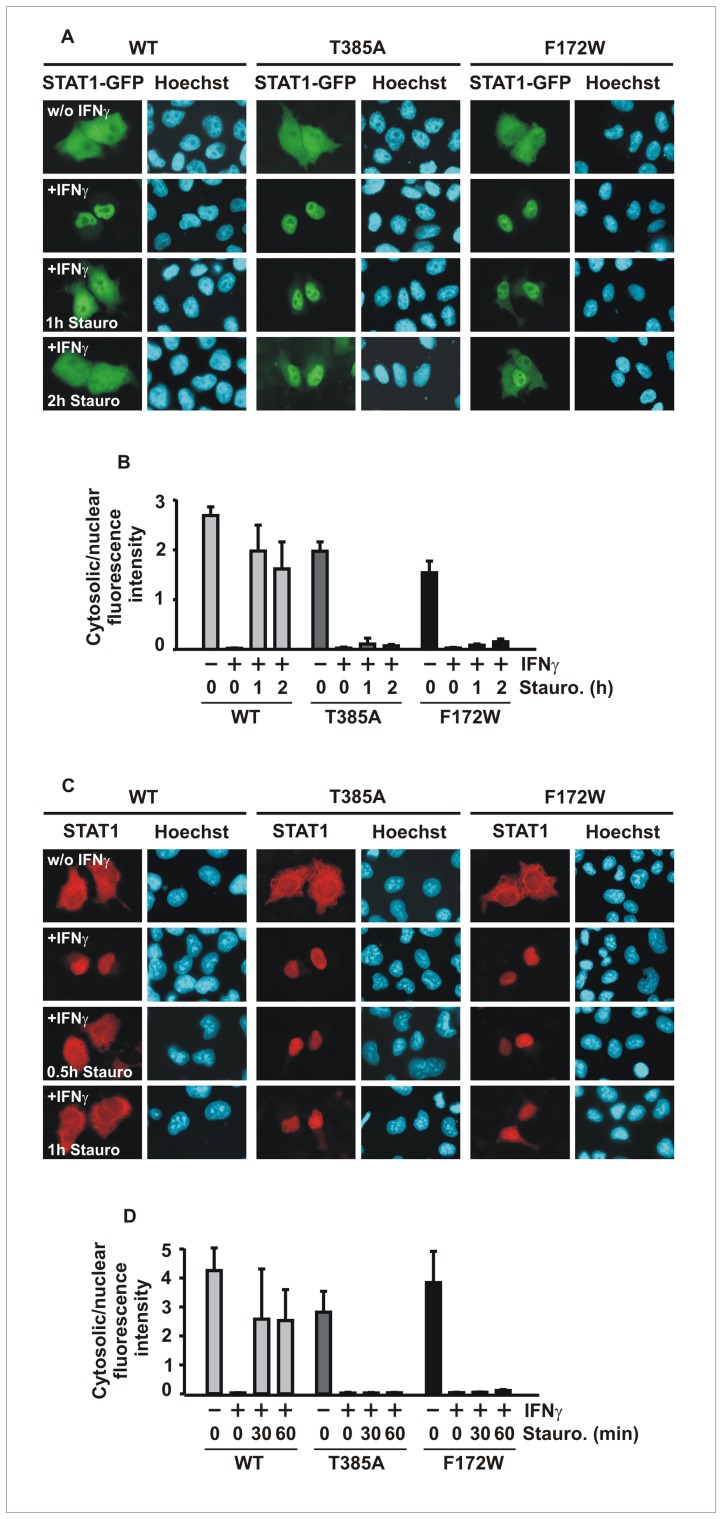
The T385A and F172W alterations are associated with prolonged interferon-γ-induced nuclear accumulation. (A, B) Within one hour of addition of staurosporine to IFNγ-pretreated HeLa cells, nuclear accumulation of GFP-tagged STAT1-WT has collapsed, while nuclear retention is unaffected by staurosporine treatment in cells expressing STAT1-T385A and -F172W. (A) Microscopic images show the intracellular localization of STAT1-GFP and the corresponding Hoechst-stained nuclei. (B) The ratios of cytoplasmic-to-nuclear fluorescence intensities in differently treated cells are depicted as means and standard deviations. (C, D) Exposure of reconstituted U3A cells to staurosporine resulted in a normal loss of cytokine-induced nuclear accumulation of untagged wild-type STAT1, but significantly retarded the nuclear residence time of mutant STAT1. (C) Immunocytochemical staining using a STAT1-specific primary and Cy3-labeled secondary antibody as well as Hoechst-stained nuclei are depicted. (D) Cytosolic/nuclear fluorescence intensities were determined and presented as histograms. All experiments were performed at least three times with similar results.

To confirm these observations, we next monitored the time course of the cytokine-induced intracellular redistribution of STAT1 using direct fluorescence microscopy. HeLa cells expressing GFP-tagged fusion proteins of STAT1 were either left untreated or stimulated for 45 min with IFNγ and consecutively exposed to staurosporine for an additional 0, 1 and 2 hours, respectively. We found that the pancellular localization in resting cells as well as the extent of nuclear accumulation in IFNγ-stimulated cells did not differ between wild-type and the mutants (Fig. 2A,B). However, when we monitored the intracellular STAT1 distribution in IFNγ-pretreated cells after addition of the kinase blocker, we observed a significant difference between the STAT1-GFP variants, as the decrease in nuclear concentration was delayed for the mutants. Similar results were also obtained, when 45 min after IFNγ-stimulation, the cytokine was washed out and replaced with control medium. Although in the absence of staurosporine it took longer for a complete loss of nuclear accumulation, the mutants still displayed a prolonged phase of nuclear retention (data not shown).

To exclude the possibility that the altered nuclear accumulation kinetics was caused by the presence of the GFP fusion protein, we performed similar experiments in reconstituted STAT1-negative U3A cells expressing the untagged STAT1 variants using immunofluorescence staining. Since Triton permeabilization of fixed cells typically removes considerable amounts of cytoplasmic soluble proteins, the time courses of STAT1 nuclear accumulation in the two experiments may not be directly comparable. Nevertheless, we confirmed that the mutants exhibited a prolonged phase of IFNγ-induced nuclear accumulation as compared to STAT1-WT (Fig. 2C, D). From these results, we concluded that the prolonged nuclear residence time of the mutants was the consequence of elevated tyrosine phosphorylation and that their hyper-phosphorylation constitutes an inherent, cell type-independent property.

### The T385A Alteration Confers Resistance to the Inhibitory Action of the Phosphatase Tc45

**Figure 3:**
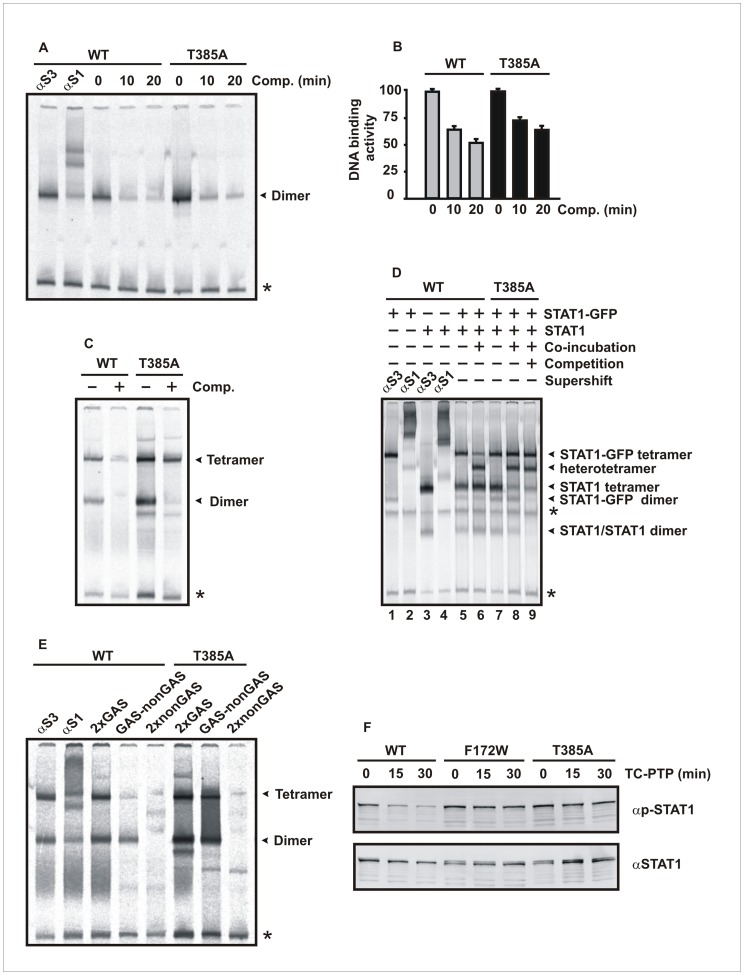
The STAT1 mutant T385A shows unaltered DNA-binding affinity to GAS elements. (A, B) Gelshift experiments demonstrated that STAT1-T385A and wild-type protein exhibit a similar dissociation rate from a single STAT1-binding site (M67). Cell lysates from IFNγ-stimulated U3A cells (5 ng/ml) expressing either wild-type or mutant STAT1 were incubated with [^33^P]-labeled M67 for 15 min and subsequently a 750-fold molar excess of unlabeled M67 was added for the durations indicated, before the reactions were loaded onto a native polyacrylamide gel. Shown is a typical gel, with the arrowhead at its right-hand margin corresponding to dimeric STAT1, including a densitometric quantification thereof. Asterisks mark unspecific bands. (C) The T385A mutant displays cooperative DNA binding due to tetramer stabilization. Extracts from an equal number of IFNγ-stimulated U3A cells expressing either wild-type or mutant STAT1 (5 µl in each lane) were incubated *in vitro* with [^33^P]-labeled DNA containing two GAS sites separated by 10 bp (2xGAS). The reactions were either left unchallenged (−) or challenged for 30 min with a 750-fold excess of a single, unlabeled GAS site (+ Competition). Note that the tetrameric, but not the dimeric occupancy of the probe resisted competition with excess unlabeled DNA. (D) Gelshift experiments demonstrate the propensity of tetrameric STAT1-T385A to exchange dimers. Shown is a representative gel using [^33^P]-labeled 2xGAS and cellular extracts from IFNγ/vanadate-co-stimulated U3A cells expressing either GFP-tagged or untagged STAT1. Supershifts with either anti-STAT3 (lane 1 and 3) or anti-STAT1 (lane 2 and 4) antibodies identified bands corresponding to DNA-bound STAT1. For the identification of dimeric STAT1 complexes, a competition experiment using 750-molar excess of unlabelled GAS was included in the last lane. Similar amounts of GFP-tagged and untagged homodimers were either immediately mixed and incubated together for 45 min (lanes 6 and 8) or incubated separately for 45 min before being loaded together onto the gel (lanes 5 and 7). Note that newly formed bands corresponding to STAT-GFP/STAT1 heterotetramers appeared only in extracts that had been co-incubated. (E) The T385A mutant binds to GAS sequences as a tetramer. Whole cell extracts from reconstituted, IFNγ-prestimulated U3A cells were incubated with various [^33^P]-labeled DNA probes containing either two (2xGAS), one (GAS-nonGAS) or no (2xnonGAS) GAS sites, respectively. In the first lane, a non-specific anti-STAT3 antibody and in the second lane, anti-STAT1 antibody C-24 was present in the EMSA reaction used for identification of STAT1-DNA complexes (marked with arrowheads). (F) The F172W and T385A mutants are defective in tyrosine dephosphorylation as revealed by an *in vitro* dephosphorylation assay using purified Tc45 phosphatase. Cell extracts from reconstituted U3A cells expressing either wild-type or mutant STAT1 (10 µl in each reaction) were incubated with 2 U of the STAT1-specific Tc45 phosphatase and tyrosine dephosphorylation was monitored with time by means of Western blotting.

In order to assess the DNA-binding kinetics of the T385A mutant, we next performed gelshift experiments using [^33^P]-labeled DNA probes and whole cell extracts from reconstituted U3A cells expressing mutant or wild-type STAT1. First, we examined the dissociation rate from a single, consensus GAS site, termed M67, and found that the off-rate of the mutant was within the same range as the wild-type protein, when challenged with a 750-fold molar excess of unlabeled GAS (Fig. 3A,B). Since defective cooperative DNA binding results in a similar phenotype with elevated IFNγ-induced tyrosine phosphorylation and prolonged nuclear accumulation [39], secondly, we examined the propensity of the T385A mutant to form stable tetramers on two GAS sites arranged in a tandem orientation (2xGAS). As shown in Figure 3C, only tetrameric, but not dimeric STAT1 complexes bound to a 2xGAS probe resisted challenge with a high molar excess of unlabeled GAS, indicating that the mutant displayed normal cooperative DNA binding. To confirm that the T385A alteration does not impact the exchange of individual dimers from STAT1 tetramers, we either co-incubated or separately exposed extracts from cells expressing either GFP- or untagged STAT1 proteins to [^33^P]-labeled 2xGAS, before they were loaded onto native polyacrylamide gels for EMSA. Both wild-type and mutant STAT1 were capable of forming heterotetrameric complexes within 45 min of co-incubation, which mainly consisted of one GFP- tagged and one untagged dimer (Fig. 3D). Thirdly, when we tested sequence requirements for DNA binding using different probes, we observed that STAT1-T385A formed tetramers on a double-stranded 45 bp oligomer with only a single GAS site flanked by non-specific sequence, termed GAS-nonGAS, confirming that the T385A alteration had no negative impact on GAS binding (Fig. 3E).

We next tested whether the hyper-phosphorylation of the F172W and T385A mutants resulted from defective dephosphorylation, as has already been assumed by Takezaki et al. and Sampaio and co-workers. For this purpose, we employed an *in vitro* dephosphorylation assay using whole cell extracts from reconstituted U3A cells (10 µl each) expressing either WT, F172W or T385A, which were incubated with 2 units of the purified, recombinantly expressed protein tyrosine phosphatase Tc45 [40]. And indeed, we found that the mutations conferred resistance against the enzymatic action of the inactivating phosphatase (Fig. 3F). Together, these results demonstrate that binding to a single GAS site was unaffected by the mutations, and that, in contrast to the wild-type protein, the mutants were protected against the catalytic attack of the STAT1-specific Tc45 phosphatase.

### Differential Regulation of Gene Expression by Mutations Located at the Dimer Interface

**Figure 4:**
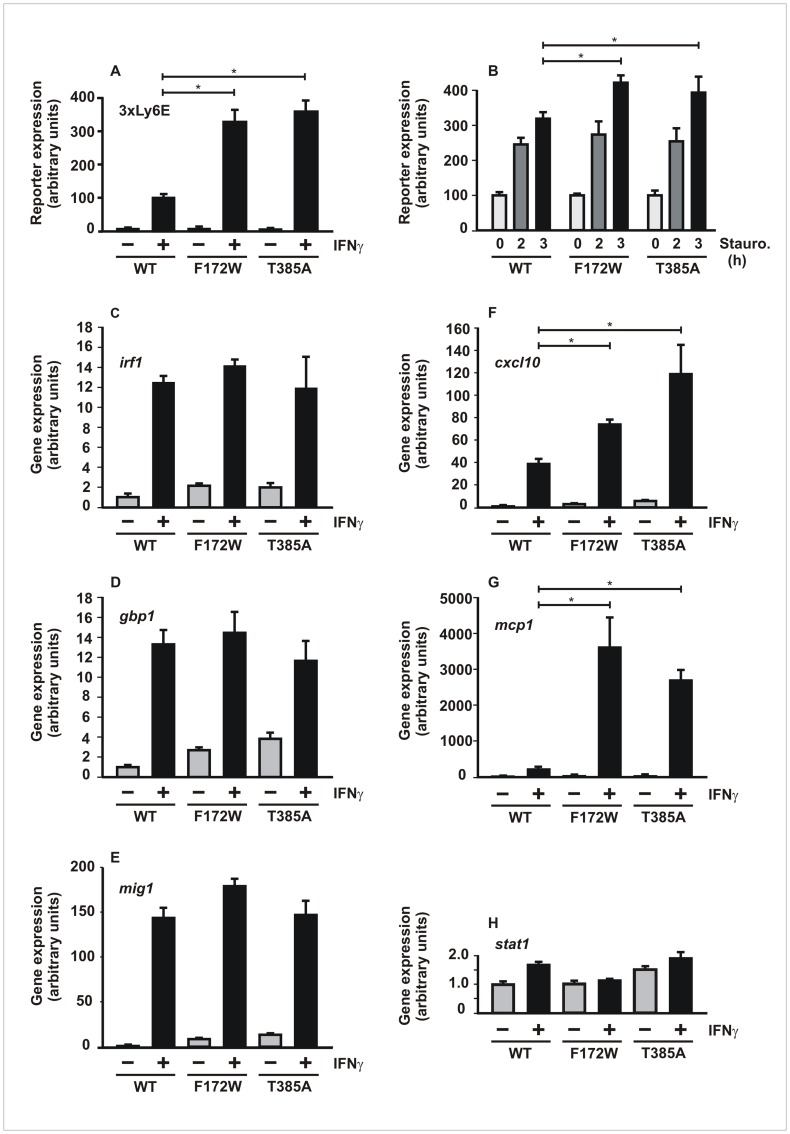
Differential gene activation by the STAT1-F172W and -T385A mutants. (A) Expression of the 3xLy6E reporter gene by mutant and wild-type STAT1 in unstimulated (gray columns) and IFNγ-stimulated (black columns) reconstituted U3A cells. (B) Inhibited down-regulation of 3xLy6E reporter expression following staurosporine treatment by the dimer interface mutants. U3A cells reconstituted with the indicated STAT1 variants were stimulated with 5 ng/ml IFNγ for 3 h and subsequently exposed to 1 µM staurosporine for 0, 2, and 3 h, respectively. Expression rates for each STAT1 variant were normalized to samples not treated with staurosporine. (C) Activation of five endogenous STAT1 target genes, and for control the transfected *stat1* gene, in U3A cells expressing either mutant or wild-type STAT1, as determined by real-time RT-PCR. Expression levels of *irf1* (C), *gbp1* (D), *mig1* (E), *cxcl10* (F) and *mcp1* (G), and for control *stat1* (H), before and 6 hours after stimulation with 5 ng/ml of IFNγ are shown. Expression data, normalized to the levels of the house-keeping gene *gapdh*, are presented as means and standard deviations. Significant differences in the IFNγ-induced gene activation between the two STAT1 variants are marked by asterisks. All experiments were repeated at least three times with similar results.

To determine whether the hyper-phosphorylated interface mutants reflected a general increase in IFNγ responsiveness, we probed the STAT1 variants for their transcriptional activity by luciferase gene assays using the 3xLy6E reporter. As shown in Fig. 4A, U3A cells expressing the indicated STAT1 variants were either left untreated (gray columns) or treated with IFNγ for 6h (black columns) before light units were counted in cellular extracts. Results showed that the two mutants were much better transcriptional activators on this luciferase construct. When transcriptional activation in IFNγ-pretreated U3A cells was halted by the addition of the potent kinase inhibitor staurosporine, the two interface mutants still showed increased reporter gene activation as compared to the wild-type protein (Fig. 4B).

We then examined a panel of previously characterized, IFNγ-induced endogenous target genes by means of real-time PCR. Unexpectedly, real-time RT-PCR showed virtually no effect of the two dimer mutations on the IFNγ-mediated induction of the *irf1*, *gbp1*, and *mig1* genes as compared to the wild-type molecule (Fig. 4C–E). However, transcriptional activity of mutant STAT1 was enhanced on the *cxcl10* and *mcp1* genes, suggesting that sequence-specific requirements account for the differential gene expression profile (Fig. 4F,G). The human *irf1*, *gbp1*, and *mig1* genes each contain a single consensus GAS-driven promoter element, whereas the *cxcl10* and *mcp1* promoters additionally contain a TTC element, 10 bp upstream from the GAS site.

**Figure 5:**
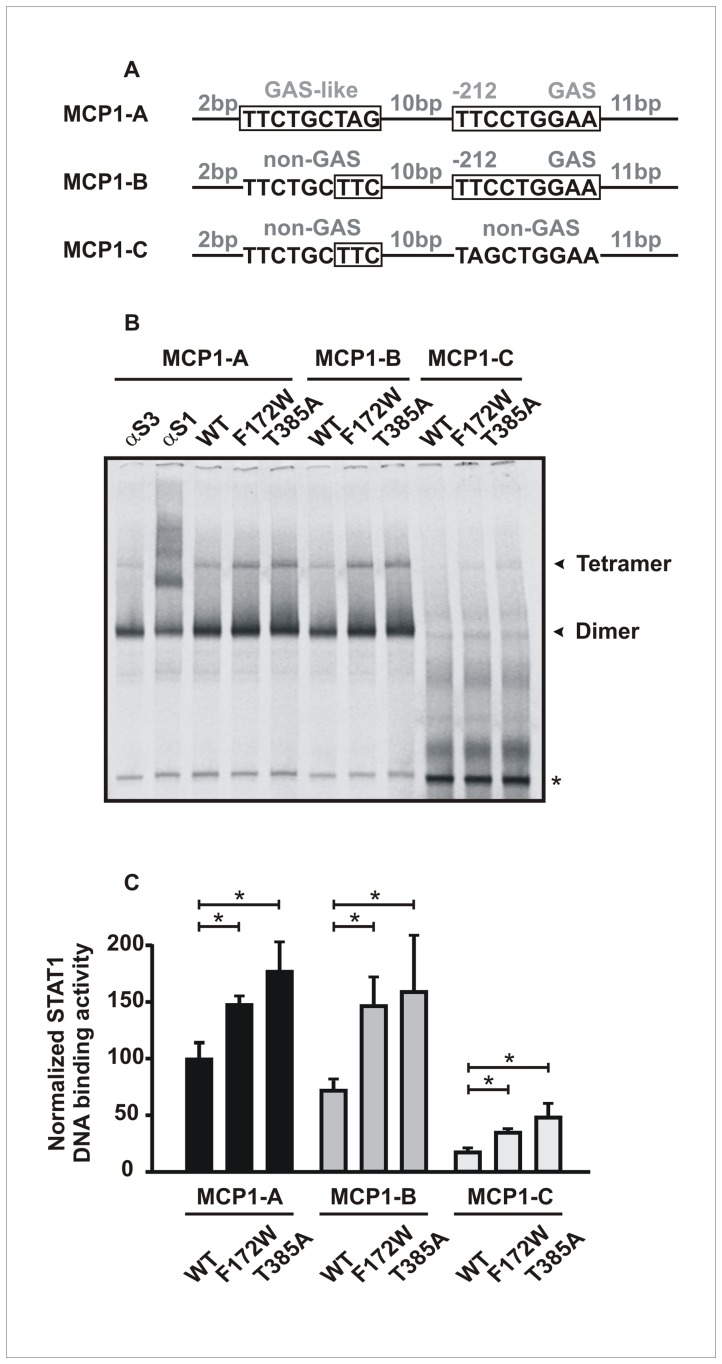
Transcriptionally active STAT1-F172W and -T385A bind to a TTC/GAA motif 10 bp upstream of a GAS site. (A–C) STAT1-T385A and -F172W, but to a much lesser extent wild-type STAT1, bound as tetrameric complexes to a [^33^P]-labeled DNA fragment from the *mcp1* promoter, which comprised a STAT1-binding site containing the one-and-a-half-GAS element. Significant binding of mutant STAT1 was observed by EMSA, when the GAS site was mutated, while leaving the TCC element intact (MCP1-C). Depicted are a drawing of the three MCP1 probes used in this study (A), a typical EMSA gel (B), and a quantification thereof showing densitometric signal intensities of tetramer-bound STAT1(C).

Thus, we wondered whether the additional presence of the TTC element adjacent to the GAS site was responsible for the distinct gene expression pattern and tested this hypothesis by studying the *mcp1* promoter in more detail. For this purpose, we mutated the native *mcp1* promoter by either introducing a second GAS-like site (MCP1-A) or disrupting the distal GAS site (MCP1-C) and analyzed the resulting native and mutated promoter constructs for binding to STAT1 (Fig. 5A). As depicted in Fig. 5B, all STAT1 variants bound as dimers to the native MCP1 probe containing the “one-and-a-half-GAS” motif (MCP1-B). The T385A and F172W mutants also showed an additional tetrameric gelshift band which, however, was rarely detectable in extracts from cells expressing wild-type STAT1, thus demonstrating the superiority of the mutants in forming stable tetramers on the native *mcp1* promoter construct. However, when the GAS site was disrupted, the proximal TTC motif being left intact, still a weak, but significant binding of mutant STAT1 tetramers was detectable.

## Discussion

Our data show that two clinically relevant interface mutants of STAT1 regulate cytokine-driven gene expression in a complex manner, depending on the presence of half a palindromic STAT1 binding element 10 bp away from a classical GAS sequence. In the absence of such a TTC/GAA binding motif, gene expression is virtually unaffected by the dimer-specific mutations, while in its presence the mutants surpass the wild-type molecule in activating IFNγ-driven target genes. The superiority of the mutants as transcriptional activators on genes containing the “one-and-a-half-GAS” motif in their promoters reflects the differential gene expression profile elicited by a structural alteration which critically destabilizes the antiparallel dimer conformation. Corroborating this observation, binding of tetrameric STAT1 to DNA was augmented in the presence of the F172W or T385A substitution, as shown by gelshift experiments using a fragment from the native *mcp1* promoter. The increased gene activation, which was observed on promoters with a TTC/GAA binding motif in the neighborhood of a consensus GAS site, appears to directly reflect the enhanced binding of the mutants to these DNA sequences. The distance of the TTC/GAA motif from the neighboring GAS site may be crucial, because the right-handed double helix of B-DNA has 10.5 base pairs per turn and may favor the formation of a tetrameric complex consisting of two dimers, which are stabilized among each other through aminoterminal interactions [41], [42]. Indeed, in gelshift experiments using DNA probes containing a “one-and-a-half-GAS” motif we found a higher degree of tetramer stabilization in the case of the dimer interface mutants.

Our data support the assumption from the Darnell laboratory that these mutants are defective in the transition from a parallel to an antiparallel dimer configuration and that the formation of antiparallel STAT1 dimers is required as a crucial step for the termination rather than the initiation of IFNγ-mediated signal transduction [31], [32]. Using recombinant Tc45 phosphatase in an *in vitro* dephosphorylation assay we demonstrate that the phenotype of the T385A mutant results from diminished enzymatic dephosphorylation, confirming that the antiparallel dimer configuration is the preferential substrate for inactivation by the phosphatase.

All STAT1 mutations, so far reported in patients with systemic or local fungal infections, are located in either the coiled-coil domain or the DNA-binding domain, which both constitute the interface surface for the antiparallel dimer configuration [9], [10], [11], [14], [15]. Due to their hyper-phosphorylation and increased responsiveness to IFNγ on a subset of genes, these disease-associated substitutions have been collectively attributed to gain-of-function mutations [9], [10], [11], [14], [15]. Although our data confirm an elevated expression rate for a subset of genes in cells expressing either F172W or T385A, we nevertheless found a substantial number of normally induced genes, all of which contained a consensus GAS site, that were grossly unaffected by the dimer-specific interface mutations. Given the key role of STAT1 in antifungal defense, it will, therefore, be important to address in more detail how these genetic alterations found in immunodeficient patients with chronic fungal infections affect target gene recognition in a genome-wide context. From our transcriptional data, we suppose that these so-called gain-of-function mutations display a rather complex behavior in terms of gene activation, which is probably beyond the categories of an overall up-regulation [43].

In summary, our data demonstrate that two disease-associated STAT1 point mutants with substitutions of residues located at the dimer interface exhibited a gene-specific transcriptional response that differed significantly from the wild-type protein. While expression levels were unaffected on genes with a single GAS site in their promoters, transcriptional activation on genes with an adjacent TTC/GAA motif at a distance of 10 bp from a GAS site was enhanced by the substitutions as compared to the wild-type protein. The molecular mechanisms behind the differential gene activation may result from facilitated cooperative DNA binding observed for mutant STAT1 tetramers, as was revealed by gelshift experiments. Furthermore, our data on the T385A mutant confirm that the antiparallel dimer is the preferential substrate for the inactivating Tc45 phosphatase. Our data are consistent with the hypotheses that a shift from a parallel to an antiparallel dimer conformation of STAT1 is required for the termination of IFNγ-mediated gene expression and that a defect in this transition causes human immunodeficiency.
